# Inhibitory Antibodies against Activin A and TGF-β Reduce Self-Supported, but Not Soluble Factors-Induced Growth of Human Pulmonary Arterial Vascular Smooth Muscle Cells in Pulmonary Arterial Hypertension

**DOI:** 10.3390/ijms19102957

**Published:** 2018-09-28

**Authors:** Tatiana V. Kudryashova, Yuanjun Shen, Andressa Pena, Emily Cronin, Evelyn Okorie, Dmitry A. Goncharov, Elena A. Goncharova

**Affiliations:** 1 Pittsburgh Heart, Lung and Blood Vascular Medicine Institute, University of Pittsburgh Department of Medicine, Pittsburgh, PA 15261, USA; tvk4@pitt.edu (T.V.K.); sheny@pitt.edu (Y.S.); azp12@pitt.edu (A.P.); dmg87@pitt.edu (D.A.G.); 2 Division of Mathematics and Sciences, Walsh University, North Canton, OH 44720, USA; ecronin1@walsh.edu; 3 Dietrich School of Arts and Sciences, University of Pittsburgh, Pittsburgh, PA 15261, USA; eco10@pitt.edu; 4 Division of Pulmonary, Allergy and Critical Care, University of Pittsburgh Department of Medicine, Pittsburgh, PA 15213, USA; 5 University of Pittsburgh Department of Bioengineering, Pittsburgh, PA 15213, USA

**Keywords:** pulmonary arterial hypertension, human smooth muscle cells, TGF-β, Activin A, Gremlin 1, therapeutic antibody, Smad proteins, PDGF-BB, growth, proliferation

## Abstract

Increased growth and proliferation of distal pulmonary artery vascular smooth muscle cells (PAVSMC) is an important pathological component of pulmonary arterial hypertension (PAH). Transforming Growth Factor-β (TGF-β) superfamily plays a critical role in PAH, but relative impacts of self-secreted Activin A, Gremlin1, and TGF-β on PAH PAVSMC growth and proliferation are not studied. Here we report that hyper-proliferative human PAH PAVSMC have elevated secretion of TGF-β1 and, to a lesser extent, Activin A, but not Gremlin 1, and significantly reduced Ser^465/467^-Smad2 and Ser^423/425^-Smad3 phosphorylation compared to controls. Media, conditioned by PAH PAVSMC, markedly increased Ser^465/467^-Smad2, Ser^423/425^-Smad3, and Ser^463/465^-Smad1/5 phosphorylation, up-regulated Akt, ERK1/2, and p38 MAPK, and induced significant proliferation of non-diseased PAVSMC. Inhibitory anti-Activin A antibody reduced PAH PAVSMC growth without affecting canonical (Smads) or non-canonical (Akt, ERK1/2, p38 MAPK) effectors. Inhibitory anti-TGF-β antibody significantly reduced P-Smad3, P-ERK1/2 and proliferation of PAH PAVSMC, while anti-Gremlin 1 had no anti-proliferative effect. PDGF-BB diminished inhibitory effects of anti-Activin A and anti-TGF-β antibodies. None of the antibodies affected growth and proliferation of non-diseased PAVSMC induced by PAH PAVSMC-secreted factors. Together, these data demonstrate that human PAH PAVSMC have secretory, proliferative phenotype that could be targeted by anti-Activin A and anti-TGF-β antibodies; potential cross-talk with PDGF-BB should be considered while developing therapeutic interventions.

## 1. Introduction

Pulmonary arterial hypertension (PAH) is a progressive and rapidly fatal disease with high mortality rates and no curative options [1,2,3,4]. In PAH, vasoconstriction of medium and large pulmonary arteries (PAs) and morphological remodeling of small PAs lead to increased PA pressure, elevated right ventricle (RV) afterload, cor pulmonale, and ultimately death [5]. Most patients with PAH are unresponsive to traditional vasodilators, and available therapies fail to reverse established pulmonary vascular remodeling or prevent disease progression, making development of effective remodeling-focused therapeutics an area of unmet important need. Increased proliferation of pulmonary arterial vascular smooth muscle cells (PAVSMC) in small PAs is a critical component of pulmonary vascular remodeling and anti-proliferative PAVSMC-focused strategies are currently under active investigation.

PAVSMCs in human PAH develop unique disease-specific hyper-proliferative phenotype, which is supported, at least in part, by dysregulation of transforming growth factor β (TGF-β) network [6,7]. TGF-β superfamily consists of nearly 30 members, including TGF-β isoforms 1, 2 and 3, bone morphogenetic proteins (BMP) and Activin A [8,9]. Most ligands of the TGF-β superfamily, except for inhibin-α, bind to type I receptors (the centerpiece) and type II receptors (the activator), which initiate Smad activation [10]. Dependent on ligand-receptor interactions, the phosphorylation of the regulated Smad (R-Smad) can transduce either TGF-β-like signals, such as the activation of Smad 2 and 3, or BMP-like signals, such as the activation of Smad1/5 [9,10]. Embryonic studies have shown that there are also several diffusible ligand-binding proteins that prevent TGF-β ligands from accessing their respective receptors, such as latency-associated protein (LAP) for TGF-β, follistatin for Activin A, and Gremlin for BMPs [10].

Compelling evidence demonstrates the importance of TGF-β axis in human PH [11]. Eighty percent of cases of familial and 20% of cases of idiopathic PAH are linked to the mutations in BMP type II receptors (BMPRII), and BMPRII dysfunction is important for the endothelial and smooth muscle cell proliferation and consequent pulmonary vascular remodeling [12,13,14]. Increased TGF-β levels are linked to hypoxia-induced PAVSMC proliferation and SU5416/hypoxia- and Schistosoma mansoni–induced pulmonary hypertension (PH) [15,16,17,18,19]. Several strategies to target TGF-β network in PAH had been developed, including selective TGF-β ligand trap to reverse PH [8], blockade of the TGF-β1-3 and its receptor to reduce Schistosoma mansoni–induced PH [19], BMPRII activation by FK506 [20], and reduction of vascular smooth muscle cell proliferation by treatment with BMP-2 agonist [21]. Comparative analysis of therapeutic attractiveness of different members of TGF-β superfamily to target hyper-proliferative PAVSMC in human PAH, however, had not been performed, and their relationship with other pro-proliferative pathways, such as platelet-derived growth factor (PDGF) signaling, known PAVSMC mitogen in PAH, remains to be established. 

PDGF-BB is a well-known growth factor that promotes PAVSMC proliferation via binding with transmembrane tyrosine kinase receptors PDGF receptor α (PDGFR-α) and PDGFR-β. That, in turn, activates multiple pro-proliferative signaling pathways [22,23,24,25,26]. PDGF-A and -B are the most prominent regulators of PAVSMCs, which express high levels of PDGFR-α and PDGFR-β [27]. Expression of PDGF and PDGFR-β is increased in lungs of PAH patients, and PDGFR inhibitor imatinib reverses experimental PH and had been tested in clinical trials for patients with PAH [6,28,29]. Importantly, PDGF and TGF-β cross-talk and regulate each other. TGF-β activates several non-canonical (Smad-independent) pathways, including p38 mitogen-activated protein kinase (MAPK), extracellular signal-regulated kinases 1/2 (ERK1/2) and phosphoinositide 3-kinase (PI3K)-Akt, which are also downstream effectors of PDGF [30]. PDGF, in turn, could activate TGF-β/Smad3 signaling [31] and cooperates with TGF-β1 to modulate low shear stress-induced aortic remodeling [16]. 

In this study, using inhibitory antibodies, we aimed to compare potential therapeutic attractiveness of scavenging major members of TGF-β superfamily, Activin A, Gremlin 1, and TGF-β1-3, on self-supported and induced growth of human PAVSMC as it relates to PAH. We report that anti-Activin A and anti-TGF-β, but not anti-Gremlin 1, antibodies significantly reduce self-supported growth and proliferation of PAVSMC from human PAH lungs while having little effect on the growth of non-diseased PAVSMC induced by soluble factors secreted by PAH PAVSMC. We also provide comparative analysis of the effects of these antibodies on canonical (Smads) and non-canonical TGF-β superfamily-dependent signaling pathways, and report that therapeutic effects of anti-Activin A and anti-TGF-β antibodies are diminished in the presence of exogenous PDGF-BB. Taken together, our data show potential attractiveness of anti-Activin A and anti-TGF-β antibodies to reduce self-sustained PAVSMC proliferation in PAH and suggest that crosstalk between TGF-β, Activin A, and PDGF pathways should be considered in future therapeutic development. 

## 2. Results

### 2.1. Human PAH PAVSMC (Pulmonary Arterial Hypertension Pulmonary Arterial Vascular Smooth Muscle Cells) Have Increased Secretion of TGF-β1 and Reduced Smad2 and Smad3 Phosphorylation Compared to Controls

**Figure 1:**
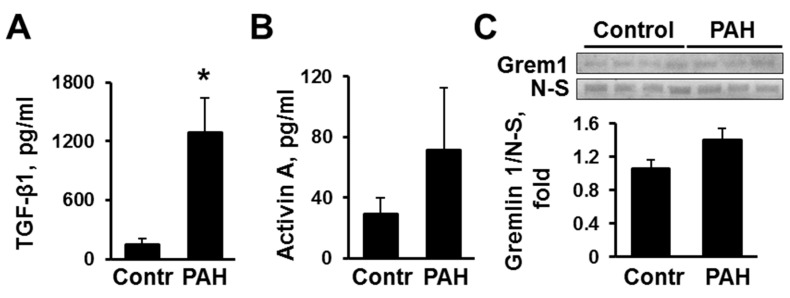
Human pulmonary arterial hypertension (PAH) pulmonary arterial vascular smooth muscle cells (PAVSMC) have increased secretion of transforming growth factor-β (TGF-β1). Human non-diseased (Control) and PAH PAVSMC were incubated for 48 h in cultural media supplemented with 0.1% bovine serum albumin (BSA); then media was collected and protein levels of TGF-β1 (**A**), Activin A (**B**), and Gremlin 1 (Grem1) (**C**) were measured in conditioned media by quantitative sandwich enzyme-linked immunosorbent assay (ELISA) (**A**,**B**) or immunoblot analysis (**C**). Data are means ± SE; * *p* < 0.05 by Mann-Whitney *U* test vs. control; *n* = 3–4 subjects/group. N-S—non-specific.

Comparative analysis of cell culture media collected after 48 h of incubation with early-passage distal human non-diseased (control) and PAH PAVSMC showed that PAH PAVSMC secrete significantly higher amounts of TGF-β1 compared to controls (1287.5 pg/mL and 147.7 pg/mL respectively), as seen in Figure 1A. The levels of Activin A were also higher in the cell culture media from PAVSMCs from patients with PAH (71.6 pg/mL vs. 29.4 pg/mL in controls), but this difference didn’t reach statistical significance, as shown in Figure 1B. In contrast, protein levels of secreted Gremlin 1 were comparable in PAH and non-diseased cells, as seen in Figure 1C. Together, these data demonstrate that human PAH PAVSMC produce higher amounts of TGF-β1 than controls, suggestive of altered TGF-β1 signaling axis.

**Figure 2:**
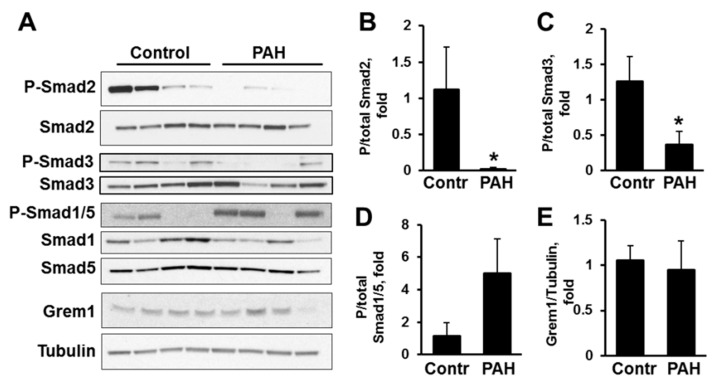
Human PAH PAVSMC have reduced phosphorylation of Smad2 and Smad3. (**A**) Human non-diseased (Control) and PAH PAVSMC were incubated for 48 h in cultural media with 0.1% BSA followed by immunoblot analysis to detect indicated proteins; (**B**–**E**) Data are means ± SE; * *p* < 0.05 by Mann-Whitney *U* test vs. control; *n* = 4 subjects/group.

Next, to evaluate the status of canonical TGF-β signaling in PAH and non-diseased PAVSMC, we tested C-terminal phosphorylation rates of Smad2 and Smad3, molecular markers of Smad activation [9]. In agreement with recently published studies, showing that Smad3 is down-regulated in PAVSMC in advanced PAH [32], we found that human PAH PAVSMC have marked reduction of active P-Ser^465/467^ Smad2 and P-Ser^423/425^ Smad3 compared to controls, as seen in Figure 2A–C. Because TGF-β and BMP pathways may reciprocally regulate each other, we next tested Smad1/5 activation status in PAVSMC from the same subjects. Interestingly, BMP-dependent Smad1/5 showed a strong trend to increased activity as evident by a marked elevation of Ser^463/465^ Smad1/5 phosphorylation in PAVSMC from 3 out of 4 analyzed PAH subjects compared to controls, as seen in Figure 2A,D. Not surprisingly, intracellular Gremlin 1 protein levels were comparable in control and PAH PAVSMC, shown in Figure 2A,E. In aggregate with our findings showing increased TGF-β1 secretion by PAH PAVSMC, these data demonstrate that Smad2 and 3 are down-regulated in human PAH PAVSMC compared to controls and suggest autocrine mechanism of Smad2 and Smad3 down-regulation due to prolonged TGF-β1 exposure.

### 2.2. Inhibitory Antibodies against Activin A and TGF-β, but Not Gremlin 1, Reduce Unstimulated Growth of Human PAH PAVSMC

**Figure 3:**
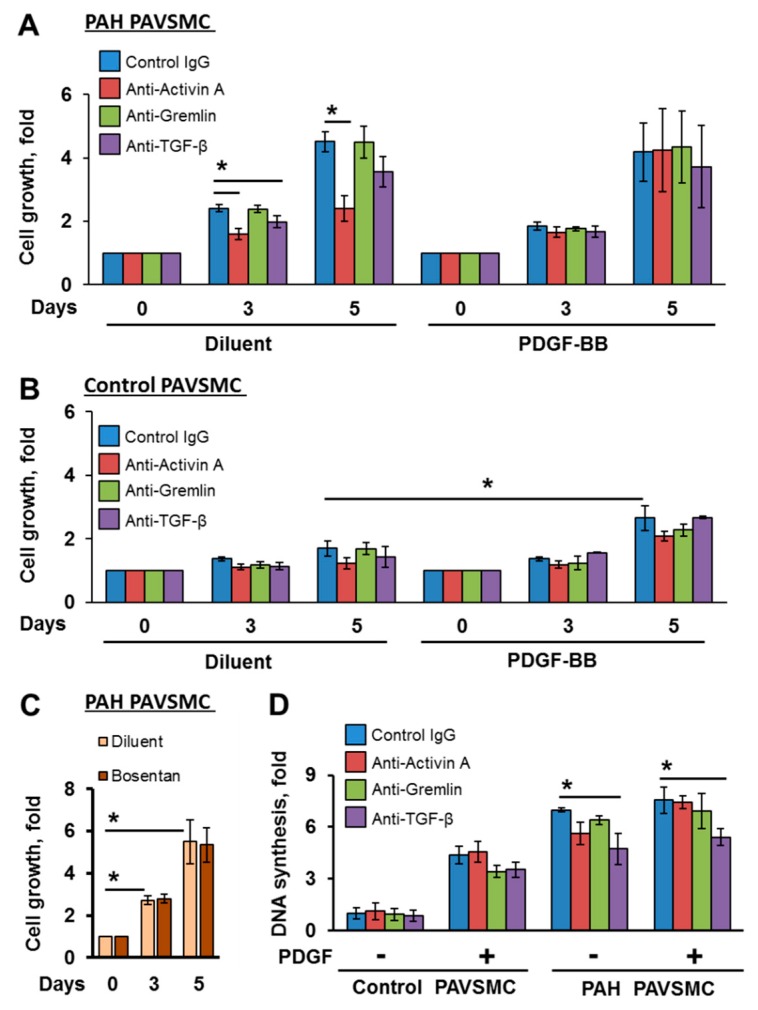
Effect of inhibitory antibodies to Activin A, Gremlin 1 and TGF-β on growth of human non-diseased and PAH PAVSMC. (**A**–**C**) Cells were maintained in changed daily serum-free media supplemented with 3.5 nM of indicated antibodies in the presence or absence (diluent) of 10 ng/mL PDGF-BB (**A**,**B**) or 1 µM bosentan or diluent (**C**); cell counts were performed at days 0, 3, and 5. Data are means ± SE representing fold to day 0 from 3 subjects/groups. (**D**) Cells, serum-deprived for 48 h, were treated with 3.5 nM of indicated antibodies in the presence or absence (diluent) of 10 ng/mL PDGF-BB for 24 h and then DNA synthesis was examined using BrdU incorporation assay. Data are means ± SE fold to control; * *p* < 0.05 by Mann-Whitney *U* test; *n* = 3 subjects/group.

Because Activin A, Gremlin 1 and TGF-β are shown to regulate cell growth and proliferation in an autocrine-dependent manner [33,34,35,36], we next evaluated an impact of therapeutic anti-Activin A, anti-Gremlin 1 and anti-TGF-β antibodies on self-supported growth of human PAH PAVSMC. In agreement with previously published studies [37,38], human PAH PAVSMC had markedly higher unstimulated growth (assessed by cell count assay) and proliferation (assessed by DNA synthesis analysis) compared to controls; PDGF-BB, while significantly promoting growth and proliferation of control PAVSMC, had little effect on PAH cells, as seen in Figure 3A,B,D. Anti-Activin A and, to a lesser extent, anti-TGF-β, but not anti-Gremlin 1 antibodies reduced self-supported growth of PAH PAVSMC, shown in Figure 3A, while bosentan, a competitive endothelin-1 receptor antagonist and a standard of care for patients with PAH, had no effect, as seen in Figure 3C. Further, anti-TGF-β antibody significantly decreased PAH PAVSMC proliferation in an absence and in presence of PDGF-BB, as seen in Figure 3D. Interestingly, PDGF-BB not only induced growth of control PAVSMC that was insensitive to TGF-β superfamily inhibitory antibodies, as shown in Figure 3B, but also diminished inhibitory effects of both, anti-Activin A and anti-TGF-β antibodies on the growth of PAH PAVSMC, as seen in Figure 3A. Together, these data suggest that Activin A and TGF-β, but not Gremlin 1, promote human PAH PAVSMC growth via the autocrine mechanism, which is in line with our findings showing increased secretion of TGF-β1 and Activin A, but not Gremlin 1, by PAH PAVSMC. These data also demonstrate growth-inhibitory effects for anti-Activin A and anti-TGF-β, but not Gremlin 1 antibody, on human PAH PAVSMC, and indicate that PDGF-BB counteracts with such inhibition, suggestive of parallel activation of pro-proliferative pathways by PDGF-BB and TGF-β.

### 2.3. Effects of Inhibitory Anti-Activin A, Anti-Gremlin 1 and Anti-TGF-β Antibodies on Canonical and Non-Canonical Downstream Targets of TGF-β Network

**Figure 4:**
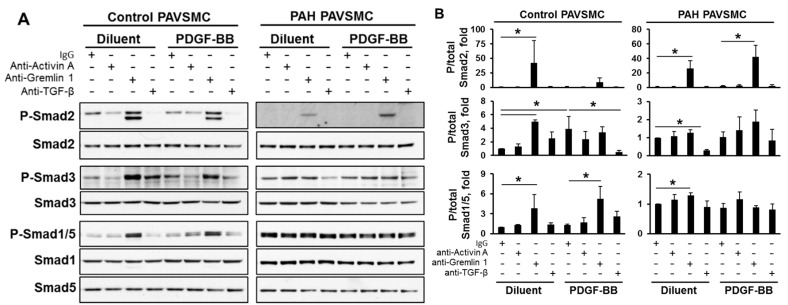
Effect of inhibitory antibodies to Activin A, Gremlin 1 and TGF-β on Smad phosphorylation status in human non-diseased and PAH PAVSMC. Cells, serum-deprived for 48 h, were treated with 3.5 nM antibodies to Activin A, Gremlin 1 and TGF-β or control IgG in the presence or absence of 10 ng/mL PDGF-BB for 18 h, and immunoblot analysis to detect indicated proteins was performed. (**A**) Representative immunoblots from three experiments, each performed on the cells from different human subject; (**B**) Data represent fold changes in P/total protein ratios with P/total ratio for control IgG without stimulation taken as 1 fold. Data are means ± SE; * *p* < 0.05 by Mann-Whitney *U* test; *n* = 3 subjects/group.

In order to understand the signaling mechanisms by which anti-Activin A and anti-TGF-β antibodies affect PAH PAVSMC growth and proliferation, we first evaluated phosphorylation status of Smads, a canonical downstream effectors of TGF-β superfamily [9]. Interestingly, treatment of control PAVSMC with PDGF-BB significantly increased Smad3 phosphorylation at the TGF-β-specific Ser^423/425^ site without affecting other Smads, as seen in Figure 4, showing that PDGF-BB may regulate Smad3 independently of TGF-β. Surprisingly, anti-Activin A antibody had no significant effect on their canonical downstream targets, Smad2 and Smad3, and did not modulate Smad1/5 phosphorylation in either PAH or control PAVSMC, as shown in Figure 4, suggesting that other mechanisms are involved. 

Interestingly, anti-Gremlin 1 antibody, while having no effect on the growth and proliferation of human PAVSMC, markedly increased not only phosphorylation of Gremlin 1 downstream effectors Smad1/5 in both, non-diseased and PAH PAVSMC, but also promoted phosphorylation of Smad2 under all tested conditions and Smad3 in diluent-treated control PAVSMC, as seen in Figure 4. 

Anti-TGF-β antibody significantly reduced Ser^423/425^ Smad3 phosphorylation rates in non-stimulated PAH PAVSMC and in PDGF-BB stimulated non-diseased cells but had little effect on Smad2 and Smad1/5 phosphorylation, seen in Figure 4. Together with our data showing that both Smad2 and Smad3 phosphorylation is already diminished in hyper-proliferative human PAH PAVSMC, shown in Figure 2, these findings failed to explain growth-inhibitory effects of anti-Activin A and anti-TGF-β antibodies in human PAH PAVSMC, as shown in Figure 3A.

**Figure 5:**
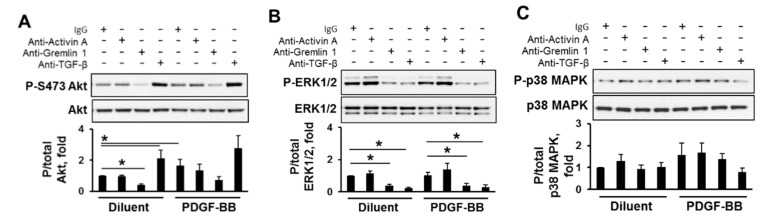
Effect of inhibitory antibodies to Activin A, Gremlin 1 and TGF-β on pro-proliferative signaling molecules in human PAH PAVSMC. Cells, serum-deprived for 48 h, were treated with 3.5 nM antibodies to Activin A, Gremlin 1 and TGF- β, or control IgG in the presence or absence of 10 ng/mL PDGF-BB for 18 h, and immunoblot analyses to detect phosphorylation status of Akt (**A**), extracellular signal-regulated kinases ½ (ERK1/2) (**B**), and p38 mitogen-activated protein kinase (MAPK) (**C**) were performed. Top panels: Representative immunoblots from three experiments, each performed on the cells from different human subject. Bottom panels: Data represent fold changes in P/total protein ratios with P/total ratio for control IgG without stimulation taken as 1 fold. Data are means ± SE, * *p* < 0.05 by Mann-Whitney *U* test; *n* = 3 subjects/group.

In addition to canonical (Smads), Activin A and TGF-β may act via non-canonical Smad-independent signaling pathways [9,10,39]. Next, we tested the effects of studied antibodies on the phosphorylation of Akt, ERK1/2 and p38 MAPK, non-canonical targets of TGF-β signaling—known pro-proliferative players in PAH pathogenesis [6,40]. We found that anti-Gremlin 1, but not anti-Activin A or anti-TGF-β antibodies reduced Akt phosphorylation, as seen in Figure 5A. Both anti-Gremlin 1 and anti-TGF-β, but not anti-Activin A, significantly decreased ERK1/2 phosphorylation in PAH PAVSMC, which was not affected by PDGF-BB treatment, as seen in Figure 5B. Interestingly, none of tested antibody modulated p38 MAPK phosphorylation rates, as shown in Figure 5C. Collectively, these data suggest that one of potential mechanisms by which anti-TGF-β antibody decrease PAH PAVSMC proliferation is via inhibition of ERK1/2 signaling pathway. The mechanism(s) by which anti-Activin A antibody reduce PAH PAVSMC growth remains to be determined.

### 2.4. Factors, Secreted by Human PAH PAVSMC, Promote Proliferation and Up-Regulate Multiple Signaling Pathways in Non-Diseased Human PAVSMC

**Figure 6:**
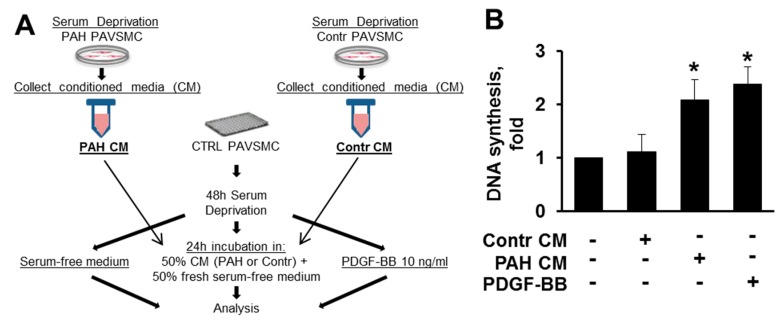
PAH PAVSMC conditioned medium promotes proliferation of non-diseased human PAVSMC. (**A**) Experimental design: Conditioned medium from serum-deprived non-diseased (Contr CM) and PAH PAVSMCs (PAH CM) was harvested after 48 h of incubation. Non-diseased cells were serum-deprived for 48 h, incubated with Contr CM or PAH CM for 24 h followed by DNA synthesis analysis using BrdU incorporation assay. Non-stimulated cells and cells treated with 10 ng/mL PDGF-BB were used as a negative and positive control, respectively. (**B**) Data are means ± SE; Data are folds to negative control; * *p* < 0.05 by Mann-Whitney *U* test vs. control; *n* = 4 subjects/group.

TGF-β and Activin A, both secreted cytokines, can bind not only to self, but also to neighboring cells to trigger respected signaling pathways [9]. To determine whether TGF-β and/or Activin A, secreted by PAH PAVSMC, induce proliferation and/or modulate signaling of non-modified PAVSMC, we incubated control PAVSMC in serum-free cell culture media conditioned by PAH (PAH CM) or control PAVSMC (Contr CM); fresh serum-free cell culture media with and without PDGF-BB were used as a positive and negative control, respectively, as shown in Figure 6A. As seen at the Figure 6B, treatment with PAH CM, but not Contr CM, significantly induced proliferation of non-diseased cells, and the magnitude of this pro-proliferative effect was comparable to proliferation induced by 10 ng/mL of well-known mitogen PDGF-BB, demonstrating that human PAH PAVSMC secrete soluble pro-proliferative factors in working concentrations.

**Figure 7:**
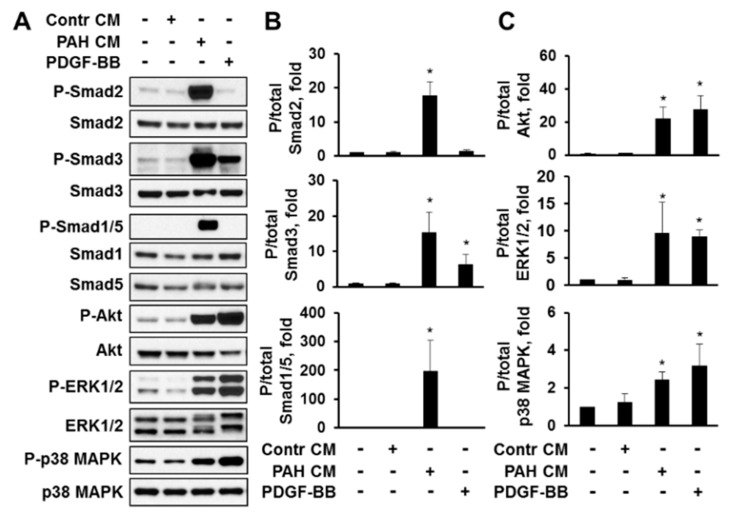
Media, conditioned by PAH PAVSMC, promotes Smad, Akt, ERK1/2 and p38 MAPK phosphorylation in non-diseased human PAVSMC. Conditioned medium from serum-deprived non-diseased (Contr CM) and PAH PAVSMCs (PAH CM) was harvested after 48 h of incubation. Non-diseased cells were serum-deprived for 48 h, incubated with Contr CM or PAH CM for 24 h, and immunoblot analysis to detect indicated proteins was performed. Non-stimulated cells and cells treated with 10 ng/mL PDGF-BB were used as a negative and positive control, respectively. (**A**) Representative immunoblots from three experiments, each performed on the cells from different human subject; (**B**,**C**) Data are means ± SE; Data are P/total ratios represented as a folds to negative control; * *p* < 0.05 by Mann-Whitney *U* test vs. control; *n* = 3 subjects/group.

Next, we performed analysis of canonical (Smads) and non-canonical downstream effectors of the TGF-β superfamily (Akt, ERK1/2, and p38 MAPK) in comparison with PDGF-BB-treated cells. We found that media, conditioned by PAH PAVSMC (PAH CM), induced significant C-terminal phosphorylation of Smad2 and Smad3 in non-diseased cells, seen in Figure 7A,B, confirming that PAH PAVSMC secrete increased amounts of active TGF-β1 and/or Activin A, shown in Figure 1. Interestingly, PAH CM also induced marked increase in phospho-Smad1/5, which are predominantly activated by BMPs as seen in Figure 7A,B. In agreement with our earlier observations shown in Figure 4, PDGF-BB increased Smad3 phosphorylation rates, but did not change the phosphorylation of Smad2 or Smad1/5, as seen in Figure 7A,B. To note, both PAH CM and PDGF-BB increased phosphorylation of Akt, ERK1/2 and p38 MAPK to a similar extent, seen in Figure 7A,C, suggesting that PAH CM could promote cell proliferation through Akt, ERK1/2 and p38 MAPK.

### 2.5. Inhibitory Antibodies against Activin A, Gremlin 1 and TGF-β Have No Effect on Proliferation of Non-Diseased PAVSMC Induced by PAH PAVSMC-Secreted Factors

**Figure 8:**
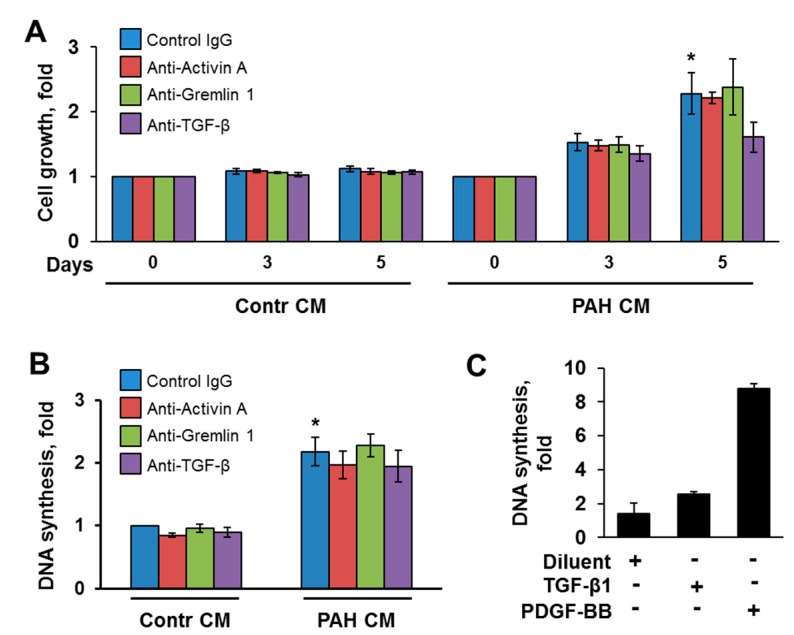
Inhibitory antibodies against Activin A, Gremlin 1 and TGF-β have no significant effect on the growth of human non-diseased PAVSMC induced by PAH PAVSMC-conditioned media. (**A**) Non-diseased (control) human PAVSMC were maintained in changed daily serum-free media supplemented with 3.5 nM of indicated antibodies in the presence of conditioned media collected after 48 h of incubation with serum-deprived control (Contr CM) or PAH PAVSMCs (PAH CM), and cell counts were performed at days 0, 3, and 5. Data are means ± SE presented as a folds to day 0; *n* = 3 subjects/group. * *p* < 0.05 by Mann-Whitney U test vs control. (**B**) Control human PAVSMC were serum deprived for 48 h, treated with 3.5 nM of indicated antibodies in the presence of Contr CM or PAH CM for 24 h, incubated with BrdU for 18 h, and DNA synthesis was examined using BrdU incorporation assay. Data are means ± SE presented as fold to control from 5 subjects/group. * *p* < 0.05 by Mann-Whitney U test vs control. (**C**) Control human PAVSMC were serum-deprived, treated with 10 ng/mL TGF-β1, 10 ng/mL PDGF-BB, or diluent for 5 days, and DNA synthesis analysis was performed using BrdU incorporation assay. Cells from two different subjects were analyzed.

To determine whether PAH PAVSMC-conditioned media induce increased proliferation of non-diseased cells via Activin A and/or TGF-β, we used inhibitory anti-Activin A and anti-TGF-β antibodies; anti-Gremlin 1 antibodies were used as additional control. As we expected, media, conditioned by PAH PAVSMC, significantly increased growth (assessed by cell count assay) and proliferation (assessed by DNA synthesis analysis) of non-diseased PAVSMC, as seen in Figure 8A,B.

Interestingly, neither anti-Activin A, nor anti-TGF-β antibodies significantly affected PAH CM-induced cell growth and proliferation, suggesting that Activin A and TGF-β have little effect on mitogen-induced growth and proliferation of human PAVSMC. To confirm our findings, we treated non-diseased human PAVSMC with 10 ng/mL TGF-β1 or PDGF-BB for 5 days to replicate the duration of cell growth experiment and performed DNA synthesis analysis. Interestingly, although PDGF-BB consistently promoted DNA synthesis in non-diseased PAVSMC, there was only a slight increase in TGF-β1-treated cells, shown in Figure 8C, suggesting that, compared to PDGF-BB, prolonged exposure to exogenous TGF-β1 has limited effect on cell proliferation. 

## 3. Discussion

Increased growth and proliferation of PAVSMC in small PAs is an important pathological component of pulmonary vascular remodeling. TGF-β superfamily plays a critical role in PAVSMC proliferation in PAH [11], but comparative analysis of its different components as molecular targets to inhibit growth and proliferation of human PAH PAVSMC had not been performed. Here, we report that proliferative distal PAVSMC derived from lungs of patients with PAH have increased secretion of TGF-β1 and, to a lesser extent, Activin A, but not Gremlin 1; factors, secreted by PAH PAVSMC are able to promote proliferation and up-regulate multiple signaling pathways in non-diseased PAVSMC. We also demonstrate that inhibitory antibodies against Activin A and TGF-β, but not Gremlin 1, reduce self-supported growth and proliferation of human PAH PAVSMC, but have no effect on proliferative response of non-diseased human PAVSMC induced by soluble factors secreted by human PAH PAVSMC.

Compelling evidence demonstrates that PAVSMC in a human PAH lung undergo complex metabolic and signaling re-programing and acquire proliferative, metabolically active phenotype with increased secretory potential [6]. Indeed, our data show that proliferative human PAH PAVSMC secrete active mitogenic factors, as evidenced by pro-proliferative effect of their conditioned media on non-diseased human PAVSMC. Supporting previously published studies [41], we found that human PAH PAVSMC secrete increased amounts of TGF-β1 and, to a lesser extent, Activin A, known regulators of proliferative response in many cell types including vascular smooth muscle cells [18,42,43,44,45,46]. Interestingly, while there is strong evidence of increased BMP antagonist Gremlin in a human PAH lung [47], we detected no differences in either endogenous or secreted Gremlin 1 between human PAH and non-diseased PAVSMC, suggesting that Gremlin 1 is produced predominantly by endothelial cells [36]. 

Canonical downstream effectors of TGF-β1 and Activin A are transcriptional factors Smad2 and Smad3 [48]. We found, however, that human PAH PAVSMC, while secreting high amounts of active TGF-β1 and/or Activin A, had reduced activatory phosphorylation of both, Ser^465/467^-Smad2 and Ser^423/425^-Smad3. These findings are in good agreement with recent reports showing marked down-regulation of Smad3 in PAVSMC from human PAH lungs and in several models of experimental PH, which appeared to be responsible for both, increased cell proliferation and reduced apoptosis [32,49]. In contrast to Smad3, down-regulation of Smad2 in PAH PAVSMC had not been reported before, and further studies are needed to dissect the mechanisms of its regulation and function in PAH. Together with published studies, our observations are suggestive of blunted Smad2 and 3 signaling in human PAH PAVSMC and may be explained by desensitization of Smad2 and Smad3 due to prolonged exposure to self-secreted TGF-β1 and Activin A, or by pathological shift from canonical Smads to non-canonical signaling pathways. 

Non-canonical TGF-β1 effectors—known regulators of vascular smooth muscle proliferation in PAH— include Akt, p38 MAPK and ERK1/2 [10,50]. It is important to note that none of those pathways are regulated solely by the TGF-β family, but act as downstream effectors of numerous pro-PAH agonists, including growth factors that signal through receptor tyrosine kinases (RTK) [6]. Further, RTK could also up-regulate Smads bypassing TGF-β receptors [51]. Because the two most common mechanisms regulating TGF-β1 signaling in the same cell are self-induced feedback loops (responsible for regulation of self-supported proliferation) and cross-talk with RTK-dependent pathways [51], we explored potential therapeutic effects of anti-Activin A, anti-Gremlin 1 and anti-TGF-β antibodies using two different scenarios, i.e., without additional exogenous stimuli and in the presence of PDGF-BB, which is up-regulated in PAH lungs, promotes PAVSMC proliferation, and activates Akt, p38 MAPK and ERK1/2 [6,26,52].

Interestingly, in contrast to the antibodies against Activin A and TGF-β, anti-Gremlin 1 antibody, while dramatically increasing phosphorylation of all tested Smads and reducing ERK1/2 phosphorylation, did not affect human PAH PAVSMC growth and proliferation. This is in good agreement with our findings showing that there are no changes in Gremlin 1 secretion by PAH PAVSMC. It should be, however, taken into account that Gremlin 1 could be secreted by and/or act through PA endothelial cells (PAEC). Indeed, recent studies from Pagano group show that Gremlin 1 modulates proliferation of PAECs in PAH [46], and Ciuclan and colleagues reported beneficial effects of antibodies against Gremlin 1 to reduce pulmonary vascular remodeling and RV pressures in mice with SU5416/hypoxia-induced PH [15], suggesting that more studies are needed to determine whether PAEC-secreted Gremlin 1 affects PAVSMC growth and proliferation in human PAH. 

We found that anti-TGF-β antibodies inhibit growth and proliferation of human PAH PAVSMC. This data supports a growing body of evidence from experimental models of PH suggesting attractiveness of TGF-β signaling as a novel molecular target pathway for therapeutic intervention in PAH [8,53,54,55]. We also report a strong inhibitory effect of anti-Activin A antibodies on increased unstimulated growth of human PAH PAVSMC. Interestingly, in contrast to anti-TGF-β antibody, which showed anti-proliferative properties, anti-Activin A antibody did not act via inhibition of proliferation, suggesting potential pro-apoptotic mechanism of action, and did not modulate either canonical Smads or non-canonical Akt, p38 MAPK and ERK1/2. In PAVSMC, Activin A can up-regulate endothelin-1 (ET-1) and plasminogen activator inhibitor-1 (PAI-1) [56], known regulator of apoptosis [57]. Given that, in our study, bosentan, selective ET-1 receptor antagonist [58,59], showed no inhibitory effect on human PAH PAVSMC growth, it would be interesting to test whether anti-growth effects of anti-Activin A antibody were due to PAI-1 regulation.

An important question remaining to be answered is whether cross-talk with growth factors, such as PDGF, should be considered before moving TGF-β1 and Activin A therapeutic antibodies to clinical studies. We found that exogenous PDGF-BB did not disturb inhibitory effects of anti-TGF-β antibody on ERK1/2 and PAH PAVSMC proliferation, but prevented anti-TGF-β and anti-Activin A antibodies-dependent reduction in cell numbers. These data, together with the well-known role of PDGF-BB as an activator of pro-survival Akt, allow us to speculate that PDGF-BB could blunt anti-growth effects of tested antibodies via promoting Akt-dependent cell survival. Another interesting observation is that neither anti-TGF-β, nor anti-Activin A antibodies were able to reduce growth and proliferation of non-diseased PAVSMC promoted by the media conditioned by human PAH PAVSMC. Such loss of effect could be explained by PAH PAVSMC-specific secretion of other pro-mitogens, which interfere with inhibitory actions of antibodies. Together, our observations allow us to hypothesize that combination therapy could be considered when targeting TGF-β signaling in PAH. 

A combination therapy is a cornerstone of anti-proliferative interventions in human cancers that provides strong anti-proliferative and/or pro-apoptotic responses via co-suppressing key pathological pathways [60]. Studies from many research groups, including ours, strongly suggest that hyper-proliferative pulmonary vascular cells in PAH share molecular similarities with cancers, which may allow application of cancer-developed therapeutic strategies to human PAH [6,50]. While further studies are needed, it is very likely that combined targeting of TGF-β-ERK1/2 and PDGF-BB-Akt axis would be beneficial to suppress PAVSMC hyper-proliferation and remodeling in PAH. Interestingly, combined inhibition of TGF-β and PDGF synergistically attenuated radiation-induced pulmonary fibrosis [61], supporting potential attractiveness of this therapeutic combination. Although pharmacological targeting of PDGFR in human PAH was associated with severe adverse events and significant side-effects [62], there are several emerging strategies to successfully block PDGF signaling either upstream (anti-PDGF antibodies [63]) or downstream of PDGFR (mTOR and Akt inhibitors) [26,37,40]. Further, TGF-β synergizes with other growth factors, including fibroblast growth factor 2, and epidermal growth factor [64,65], which could also be considered as potential molecular candidates for developing anti-TGF-β-based combination therapies. 

In conclusion, our study provides important information about the potential therapeutic attractiveness of antibodies against TGF-β and Activin A, but not Gremlin 1, to inhibit self-supported growth and proliferation of human PAH PAVSMC. We realize, however, that this study has several limitations. Although performed on primary human cells, a “gold standard” in vitro model for translational and mechanistic research focused on human PAH-related PAVSMC pathogenesis, we have tested only one type of cell, and additional studies are needed to test those antibodies on other pulmonary vascular cells, such as PAECs and PA adventitial fibroblasts. Another limitation is that this work is performed in vitro, and further testing of these agents using in vivo models of experimental PH would be needed to evaluate it efficiency at the organismal level. Last, we evaluated only few members of the TGF-β superfamily. Interestingly, while inducing strong Smad2 and Smad3 phosphorylation in non-diseased cells, cell culture media, conditioned by PAH PAVSMC, also promoted significant Smad1/5 phosphorylation. This data suggest potential involvement of BMPs in self-supported PAH PAVSMC proliferation and call for further studies to evaluate BMP-Smad1/5 interactions in human PAH PAVSMC. 

## 4. Materials and Methods

### 4.1. Human Cell Cultures

Primary distal PAVSMCs from patients with PAH and non-diseased lungs were provided by the University of Pittsburgh Vascular Medicine Institute Cell Processing Core under protocols approved by University of Pittsburgh Institutional Review Board (see Table 1 for the subject’s characteristics). Cell isolation, characterization, and maintenance were performed as described in [37]; we followed the recent recommendations for PAH preclinical research published in [66,67] as it relates to exploratory in vitro studies. Experiments were performed on primary (3–8 passage) PAVSMCs. Cells were maintained in complete LONZA growth media with SMGM-2 supplement, 100 U/mL penicillin, and 0.1 mg/mL streptomycin (Lonza Group, Basel, Switzerland). Prior DNA synthesis and immunoblot analysis experiments, cells were incubated for 48 h in basal media supplemented with 0.1% bovine serum albumin (BSA).

### 4.2. Analysis of TGF-β, Activin A and Gremlin 1 Secretion

Conditioned media was collected from serum-deprived cells after 48 h of incubation. Secretion of TGF-β1 and Activin A was evaluated by quantitative sandwich ELISA (BMS249/4, affymetrix, eBioscience, Santa Clara, CA, USA; ab113316, Abcam, Cambridge, MA, USA, respectively). Secretion of Gremlin 1 was evaluated by immunoblot analysis with specific antibody (Thermo Fisher Scientific PA5-13123).

### 4.3. Inhibitory Antibodies

Inhibitory antibodies against TGF-β (clone 1D11) were purchased from BioXcell (BE0057); inhibitory antibodies against Gremlin 1, Activin A, and control IgG were generously provided by Regeneron Pharmaceuticals. Working concentrations of antibodies were locked on 3.5 nM based on previously published studies [19] and our pilot experiments with anti-Activin A antibody (Figure S1). Briefly, working concentration was calculated as [IC50 of Activin A-dependent Smad2 and 3 phosphorylation] × 10 and verified on human non-diseased and PAH PAVSMC by analysis of inhibitory effects of 3.5 nM anti-Activin A antibody on Activin A-dependent Smad2, Smad3, and Ser^473^Akt phosphorylation (Figure S1).

### 4.4. Cell Growth and Proliferation Assays

Cell growth analysis was performed using cell counts assay as described previously [26,37,38]. Briefly, equal quantities of cells (300,000 cells/well) were plated on 6-well plates. In 48 h, cells were serum-deprived and maintained in daily-changed serum-free media supplemented with 3.5 nM inhibitory antibodies against TGF-β, Gremlin 1, Activin A, or control IgG in the presence of human recombinant PDGF-BB (10 ng/mL) or diluent. In parallel experiments, cells were plated as described above, and then incubated with 1 µM bosentan or diluent; or with cell culture media, conditioned for 48 h by human PAH PAVSMC or human control PAVSMC (filtered and mixed 1:1 with fresh serum-free media). Cell counts were performed at days 0 (48 h after plating), 3, and 5 using Countess^TM^ II FL cell counting system (Invitrogen, Grand Island, NY, USA). Experiments were repeated on the cells from a minimum of 3 subjects/group; three separate measurements per each condition in each experiment were performed. 

Cell proliferation was assessed by DNA synthesis analysis using bromodeoxyuridine (BrdU) incorporation assay according to manufacturer protocol (Cell signaling Technology, Danvers, MA, USA); normalization to cell numbers using crystal violet staining was performed. For antibody testing experiments, pre-confluent cells were serum-deprived for 48 h, treated with 3.5 nM antibodies to TGF-β, Gremlin 1, Activin A, or control IgG in the presence or absence of human recombinant PDGF-BB (10 ng/mL) for 24 h, incubated with BrdU for 18 h, and BrdU incorporation assay was performed. Experiment was performed on the cells from 3 subjects/group. To determine the effect of conditioned media on the proliferation of non-diseased (control) PAVSMC, the media was harvested from both, control and PAH PAVSMC (serum-deprived; incubated with serum-free media for 48 h). Collected media was filtered and mixed with equal amount of fresh serum-free media (1:1). The mixture was added to the serum-deprived PAVSMC from 4 different subjects. Cells incubated in 100% fresh serum-free media in the absence or in presence of 10 ng/mL PDGF-BB were used as negative and positive controls, respectively. After 24 h of incubation, cell proliferation was examined using BrdU incorporation assay. To estimate the proliferation of control PAVSMC under human recombinant TGF-β1 (10 ng/mL) or PDGF-BB (10 ng/mL) cells from 2 non-diseased subjects were serum-deprived, treated with indicated factors or diluent for 5 days, and DNA synthesis analysis was performed.

### 4.5. Immunoblot Analysis

Immunoblot analysis was performed as described before [37,38,40]. All antibodies (excluding anti-Gremlin 1) were purchased from Cell Signaling Technology; anti-Gremlin 1 antibody was purchased from Thermo Fisher Scientific. For comparative analysis PAVSMC from 4 non-diseased (control) and 4 PAH subjects were collected in cell lysis buffer after 48 h of serum deprivation and immunoblot analysis was performed. For therapeutic antibody testing experiments, cells, serum-deprived for 48 h, were pre-treated with 3.5 nM inhibitory antibodies to TGF-β, Gremlin 1 and Activin A for 30 min and then PDGF-BB (10 ng/mL) or diluent was added. After 18 h of incubation, whole cell protein was extracted and immunoblot analysis with specific antibodies against P-Smad2 (CS3108), Smad2 (CS5339), P-Smad3 (CS9520), Smad3 (CS9523), P-Smad1/5 (CS9516), Smad1 (CS6944), Smad5 (CS12534), P-Akt (CS4060), Akt (CS9272), P-p38 MAPK (CS4511), p-38 MAPK (CS8690), P-ERK1/2 (CS4377), ERK1/2 (CS4695), α/β Tubulin (CS2148) was performed and phospho/total ratios were calculated. Experiments were repeated on the cells from 3 non-diseased (control) and 3 PAH subjects. For analysis of the effect of conditioned media, conditioned media from serum-deprived non-diseased (Contr CM) and PAH PAVSMC (PAH CM) was collected after 48 h of incubation. Non-diseased cells from 3 subjects were serum-deprived for 48 h, incubated with Contr CM, PAH CM (filtered and mixed 1:1 with fresh serum-free media) or 10 ng/mL PDGF-BB for 24 h, lysed, and immunoblot analysis was performed.

### 4.6. Statistical Analysis

Immunoblots were analyzed using ImageJ (NIH, Bethesda, MD, USA), and StatView (SAS Institute, Cary, NC, USA) software. Statistical comparisons between two groups were performed by the Mann-Whitney *U* test. Statistical significance was defined as a *p* value less than or equal to 0.05.

## Figures and Tables

**Table 1:** Human subjects’ characteristics.

Age	Gender	Diagnosis
40	F	Non-diseased
64	F	Non-diseased
38	F	Non-diseased
33	F	Non-diseased
37	M	Non-diseased
40	F	IPAH
53	F	IPAH
21	M	IPAH
45	M	PAH
50	F	IPAH

F—female; M—male; IPAH—idiopathic pulmonary arterial hypertension; PAH—pulmonary arterial hypertension.

## Supplementary Materials

Supplementary materials can be found at http://www.mdpi.com/1422-0067/19/10/2957/s1.

## Abbreviations

BMP	Bone morphogenetic protein
BrdU	Bromodeoxyuridine
BSA	Bovine serum albumin
CM	Conditioned medium
ELISA	Enzyme-linked immunosorbent assay
ERK	Extracellular signal-regulated kinases
ET-1	Endothelin-1
LAP	Latency-associated protein
MAPK	Mitogen-activated protein kinase
PA	Pulmonary artery
PAEC	Pulmonary arterial endothelial cells
PAH	Pulmonary arterial hypertension
PAI-1	Plasminogen activator inhibitor-1
PAVSMC	Pulmonary artery vascular smooth muscle cell
PDGF	Platelet-derived growth factor
PDGFR	Platelet-derived growth factor receptor
PI3K	Phosphoinositide 3-kinase
R-Smad	Regulated Smad
RTK	Receptor tyrosine kinases
RV	Right ventricle
TGF-β	Transforming growth factor β
