# Supplementary material for: Inhibitory Antibodies against Activin A and TGF-β Reduce Self-Supported, but Not Soluble Factors-Induced Growth of Human Pulmonary Arterial Vascular Smooth Muscle Cells in Pulmonary Arterial Hypertension
Source: Int J Mol Sci. 2018 Sep 28;19(10):2957. doi: 10.3390/ijms19102957 (PMC6212879; doi:10.3390/ijms19102957)
Supplement: Supplementary file 1 [file ijms-19-02957-s001.zip › ijms-356272-supplementary.pdf]

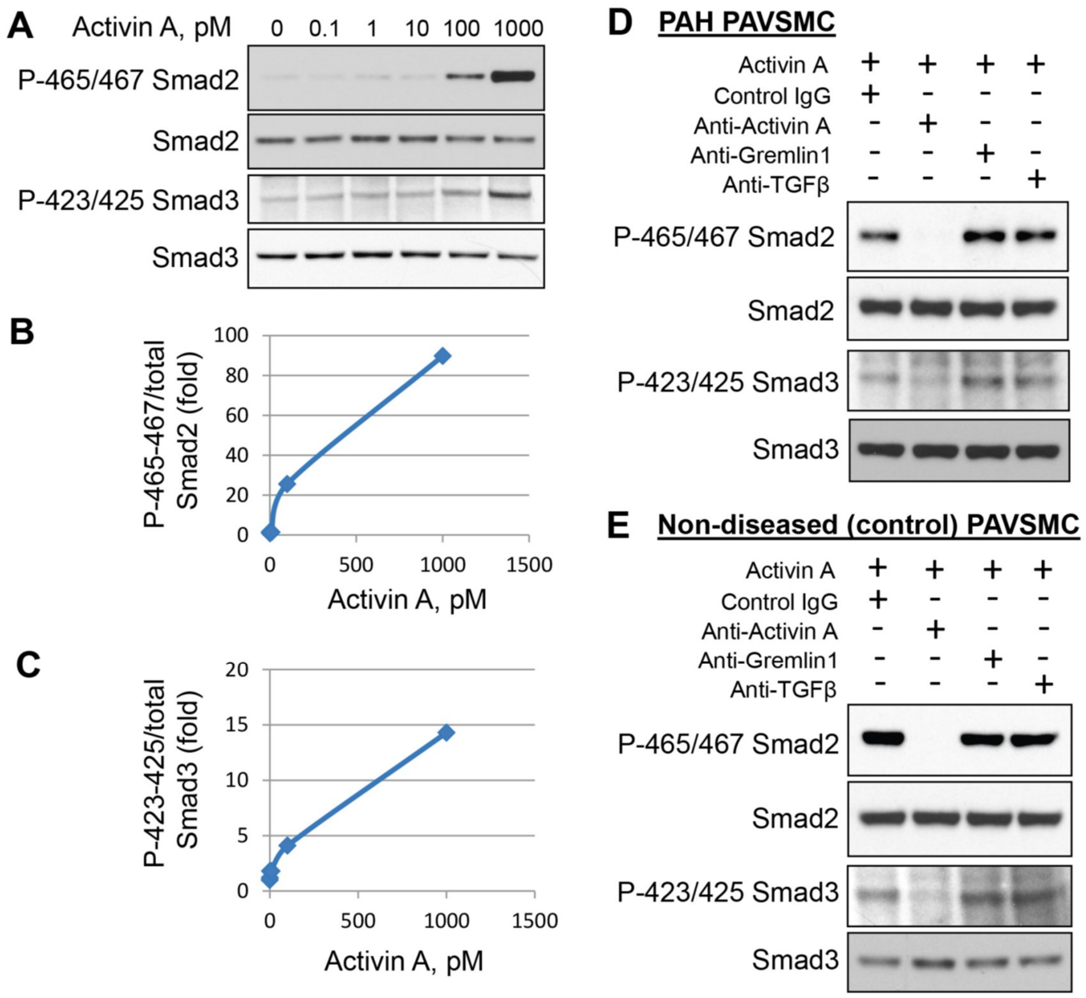

Figure S1. Establishing working concentration for inhibitory anti-Activin A antibody for human PAVSMC. A-C: Analysis of Smad2 and Smad3 phosphorylation in PAH PAVSMC induced by Activin A. (A) Human PAH PAVSMC were serum deprived for 48h in cultural media supplemented with 0.1% BSA, treated with indicated concentrations of Activin A for 18 h and then subjected to immunoblot analysis to detect indicated proteins. The EC50 value of Activin A on Smad2 phosphorylation at S465/467 (B) and on Smad3 phosphorylation at S423/425 (C) was calculated as ~350pM. Based on this, concentration of therapeutic antibodies in further experiments had been fixed at 3500 pM (3.5 nM). D-E: Inhibitory antibodies to Activin A selectively reduce Activin A-dependent Smad2 and Smad3 phosphorylation in PAH (D) and non-diseased (Control) human PAVSMC (E). PAVSMC were serum-deprived for 48 h, treated with 3.5 nM antibodies to Activin A, Gremlin 1 and TGF-β or control IgG in the presence of 350 pM recombinant Activin A. After 18 h of incubation, immunoblot analysis with indicated antibodies was performed.
